# Maternal periconception food insecurity and postpartum parenting stress and bonding outcomes

**DOI:** 10.3389/fnut.2024.1275380

**Published:** 2024-02-26

**Authors:** Karina M. Shreffler, Caitlin M. Dressler, Lucia Ciciolla, Marianna S. Wetherill, Julie M. Croff

**Affiliations:** ^1^Fran and Earl Ziegler College of Nursing, University of Oklahoma Health Sciences, Oklahoma City, OK, United States; ^2^Department of Psychology, Oklahoma State University, Stillwater, OK, United States; ^3^Department of Health Promotion Sciences, University of Oklahoma Tulsa Schusterman Center, Tulsa, OK, United States; ^4^Department of Family and Community Medicine, University of Oklahoma Tulsa Schusterman Center, Tulsa, OK, United States; ^5^Department of Rural Health, Oklahoma State University Center for Health Sciences, Tulsa, OK, United States

**Keywords:** food insecurity, first 1000 days, pregnancy, maternal, parenting stress, bonding

## Abstract

Food insecurity during pregnancy is associated with various adverse pregnancy outcomes for the mother and infant, but less is known about the role of periconception food insecurity and its links to maternal and child wellbeing in the postpartum period. In a sample of 115 diverse (41% white) and predominately low-income mothers, results of hierarchical regression analyses showed that periconception food insecurity was positively associated with parenting stress at 2 months postpartum. A negative association between food insecurity and maternal–infant bonding at 6 months postpartum was mediated after controlling for prenatal depression, social support, and demographic factors. Findings highlight the need for maternal linkage to effective food security programs, such as United States-based Special Supplemental Nutrition Program for Women, Infants, and Children (WIC), for women during their childbearing years due to the critical importance of food security for maternal and infant well-being.

## Introduction

Approximately 11% of households in the U.S. report food insecurity (1), defined as a limited availability of nutritionally adequate and safe foods or the ability to acquire such foods in a socially acceptable way (2). Food insecurity during pregnancy is associated with many negative health outcomes for the mother and infant (3), including increased maternal stress and depression (4, 5), as well as iron deficiency which potentially could lead to developmental concerns in the neonate (6). Food insecurity during pregnancy has also been associated with increased risk of birth defects (7) and poor health outcomes for the infant in the short term as well as throughout childhood (3, 8, 9). As a result of these, and other studies, the American Academy of Pediatrics (10) issued a policy statement endorsing food insecurity screening in routine clinical practice. Less is known, however, about the role of food insecurity during the periconception period on future maternal-child indicators of wellbeing, which has important implications for future policy guidance for reducing risk prior to conception.

The consequences of periconception food insecurity for maternal and infant adverse pregnancy outcomes due to nutritional deficiencies are well-known (11). Existing programs such as the federal food program offered through the Special Supplemental Nutrition Program for Women, Infants, and Children (WIC) target the reduction of maternal food insecurity during pregnancy in an effort to ameliorate the adverse nutritional impacts of food insecurity on maternal and infant health well-being. Food insecurity also has the potential to influence maternal and infant health and well-being as it is stressor that has been linked to a variety of adverse mental and behavioral health outcomes (12, 13). In an adaptation of the Family Stress Model (14), Ashiabi and O’Neal (15) proposed that food insecurity adversely affects children’s outcomes through compromised parenting associated with elevations in parental stress and mental health problems. The maternal–infant relationship begins to form early in pregnancy and continues to develop through the pregnancy, during the immediate postpartum period, and throughout early infancy and childhood (16). Maternal–infant bonding is vital for the health and well-being of both the mother and infant (17–19). Parenting stress, on the other hand, arises when mothers report difficulties adapting to the demands of parenting (20) and is considered a key determinant of subsequent parenting quality and behaviors and as well as adverse child developmental outcomes (21). Although the nutritional consequences of perinatal food insecurity are well-known, there is a lack of data examining causal stressor-related impacts of maternal food insecurity. Understanding the implications of periconception maternal food insecurity for key aspects of the maternal–infant relationship—parenting stress and bonding—is critical. Food insecurity is modifiable by interventions through programs aimed at easing this burden for families, and as such makes periconception food insecurity a potential target for such interventions. The current study fills a gap in the literature by exploring the association between periconception food insecurity and parenting stress at 2 months postpartum and the mother-infant bond at 6 months postpartum. Depressive symptoms are included in the study as a control variable due to the strong associations between depression and a variety of parenting outcomes including stress and bonding (22). Social support is included as a control variable as well because prior studies indicate it can mediate early pregnancy stressors on maternal–infant wellbeing in the postpartum period (23). We expect to find that reported food insecurity in the year preceding the first prenatal appointment will be associated with worse mother-infant relational outcomes in the postpartum period. We also expect to find that psychosocial factors of maternal depression and social support will mediate the association between periconception food insecurity and postpartum parenting stress and bonding outcomes.

## Methods

### Sample

Data for the present study were collected as part of a clinic-based longitudinal cohort study. The cohort study included 177 pregnant women (ages 16–38) that were recruited at their first prenatal appointment during 2017 and 2018 from two urban perinatal clinics in a south-central U.S. state. IRB approval was obtained prior to data collection. Nurses screened potential participants, and research team members reviewed the study procedures and ensured written informed consent/assent was collected before study participation could begin. To be eligible for the study, participants had to be able to participate in English or Spanish and had to be planning to give birth and parent the child (i.e., participants were not eligible if they planned to have an abortion or place the baby for adoption as a goal of the study was to follow participants and their children into the postpartum period). Recruitment sites serve a racially diverse and primarily socioeconomically disadvantaged patient population; approximately 90% of study participants reported receiving public insurance and the majority of participants (59%) reported racial/ethnic minority group identity. The present study included 115 of the original 177 participants who responded from the first trimester of pregnancy through the sixth wave of data collection, occurring at 6 months postpartum.

### Measures

Parenting stress was measured using the 4-item Parenting Stress Index (PSI) (24) at approximately 2 months postpartum, with higher values indicating greater stress, with a range of 4 through 19 and Cronbach’s alpha of 0.71, indicating acceptable reliability. At the six-month postpartum assessment, postpartum bonding was measured using the 24-item Postpartum Bonding Questionnaire (PBQ) (25). Responses were coded from 0 to 5 such that higher values indicated greater bonding, with a range of 58 to 129 and Cronbach’s alpha of 0.91 in the current sample, indicating high reliability.

At the first assessment, food insecurity over the previous 12 months was measured using the United States Department of Agriculture (USDA) 6-item short form, and participants were categorized into four groups: high food secure (0 points), marginal food secure (1 point), low food secure (2–4 points), and very low food secure (5–6 points) (26). Maternal depressive symptoms were assessed in the third trimester using the 20-item Center for Epidemiologic Studies Depression (CES-D) scale (27) and coded and summed to create a scale with a range of 0–47 and Cronbach’s alpha reliability of 0.89 in the current sample. Social support was measured using the Multidimensional Scale of Perceived Social Support (28) and coded and summed to create a scale with a range of 12–84 and Cronbach’s alpha reliability of 0.96 in this sample. Demographic variables included in the study were race/ethnicity coded into dummy variables using the Census priority coding scheme for White, Black, Hispanic, American Indian, and “others.” Education was included as a continuous variable for years, living in a married or cohabiting union was included as a dichotomous variable, and parity was included as a continuous variable ranging from 0 to 9 or more children.

### Analysis

Descriptive statistics were calculated for the study. Hierarchical linear regression conducted in Statistical Package for the Social Sciences (SPSS v27.0) was used to examine the associations between study variables, adjusting for demographic characteristics. Model 1 includes food insecurity categories with “high food security” as the reference category. Model 2 includes psychosocial factors of depressive symptoms and social support, as well as sociodemographic covariates, including race, education status, married or cohabiting union status, and parity. Materials and analysis code for this study are available by emailing the corresponding author.

## Results

See Table 1 for affirmative responses to each item of the food insecurity assessment. Nearly half of participants (49%) reported high food security, with 18% reporting marginal food security, 21% reporting low food security, and 12% reporting very low food security in the year preceding pregnancy (see Table 2 for descriptive statistics of study variables). The mean value of parenting stress measured at 2 months postpartum fell near the midpoint of the scale (*M* = 9.12, *SD* = 3.31), whereas the average bonding score reported at 6 months postpartum was closer to the higher end of the scale range (*M* = 122.70, *SD* = 10.34).

**Table 1:** Affirmative responses to food insecurity measure.^a^

In the past 12 months	*%*
The food I bought did not last, and I did not have money to buy more.	38%
I could not afford to eat balanced meals.	34%
I cut the size of my meals or skipped meals because there wasn’t enough money for food.	17%
How often did this happen?^b^	13%
I ate less than I felt I should because there wasn’t enough money for food.	14%
I was hungry but did not eat because there wasn’t money for food.	15%

^a^Six-item short form of the USDA Food Security Survey; ^b^Item only asked of participants who affirmatively responded to prior question; almost every month or some months but not every month = 1.

**Table 2:** Descriptive statistics of study variables (*N* = 115).

Variables	*M* or %	SD
*Food insecurity (1st trimester)*		
High food security	49%	
Marginal food security	18%	
Low food security	21%	
Very low food security	12%	
*Outcome variables*		
Parenting stress (2 mo. postpartum)	9.12	3.31
Bonding (6 mo. postpartum)	122.70	10.34
*Psychosocial variables*		
Depressive symptoms (3rd trimester)	14.96	9.85
Social support (2nd trimester)	65.46	18.59
*Sociodemographic control variables*		
Race/ethnicity		
White (reference)	41%	
Black	27%	
Hispanic	13%	
American Indian	18%	
Other race	1%	
Education (in years)	12.98	1.95
Married or cohabiting	61%	
Parity	1.30	1.49

To examine the association between food insecurity in the 12 months prior to the participants’ first prenatal visit, parenting stress 2 months after giving birth, and self-reported postpartum bonding approximately 6 months after giving birth, hierarchical regression analyses were conducted (see Table 3). Results showed that having very low food security during the first trimester predicted parenting stress at 2 months postpartum (*B* = 3.69; *p* < 0.01) and that the association remained significant after controlling for depressive symptoms (*B* = 0.09, *p* < 0.05), social support (*B* = −0.04, *p* < 0.05) and sociodemographic variables. We also found that periconception food insecurity was associated with lower self-reported postpartum bonding at the 6-month postpartum assessment in Model 1; low food security (*B* = −7.85, *p* < 0.01) and very low food security (*B* = −7.35, *p* < 0.05) predicted maternal–infant bonding at 6 months postpartum. After controlling for psychosocial and sociodemographic factors in Model 2, however, the association between food insecurity and bonding was no longer significant, suggesting a mediating effort of social support (*B* = 0.17, *p* < 0.01).

**Table 3:** Hierarchical regression analysis of maternal parenting stress (2 months) and postpartum bonding (6 months) by pre-pregnancy food insecurity, depressive symptoms, and demographic characteristics (*N* = 115).

	*Parenting stress*				*Postpartum bonding*			
	Model 1		Model 2		Model 1		Model 2	
*Variables*	*B*	*SE*	*B*	*SE*	*B*	*SE*	*B*	*SE*
*Food insecurity*								
High food security (ref)								
Marginal food security	−0.57	0.92	−1.07	0.95	−1.85	2.80	−0.86	2.89
Low food security	0.67	0.82	−0.45	0.88	−7.85**	2.49	−3.96	2.68
Very low food security	3.69**	1.10	3.27**	1.12	−7.35*	3.36	−5.18	3.41
*Psychosocial variables*								
Depressive symptoms^a^			0.09*	0.03			−0.16	0.11
Social support^b^			−0.04*	0.02			0.17**	0.06
Race/ethnicity								
White (reference)								
Black			−0.33	0.79			−1.84	2.41
Hispanic			0.90	0.93			−0.63	2.83
American Indian			−0.65	0.89			1.51	2.71
Other race			−0.70	2.23			4.02	6.81
Education (in years)			0.24	0.17			−0.42	0.53
Married or cohabiting			0.66	0.73			−5.31*	2.22
Parity			−0.28	0.21			−0.76	0.63
Constant	8.51***	0.46	6.83*	2.88	125.85***	1.41	125.60***	8.80
Adj. *R*^2^	0.10		0.21		0.09		0.20	

***p < 0.001; **p < 0.01; *p < 0.05. ^a^Measured in the 3rd trimester; ^b^measured in the 2nd trimester. Food insecurity and socio-demographic variables were measured in the 1st trimester.

## Discussion

The results of the present study suggest that food insecurity during the periconception period has the potential to impact the maternal–infant relationship in the short and long-term postpartum period. In particular, participants with very low food security reported significantly greater parenting stress and lower levels of maternal–infant bonding as compared to participants with high food security. The current study therefore provides support for Ashiabi and O’Neal’s (15) adaptation of the Family Stress Model and suggests that these negative impacts on parental well-being and the parent–child relationship can emerge very early in the infant’s life, setting the stage for long-term developmental risk (16). On the other hand, the findings also reveal that perceived social support during pregnancy can mediate the adverse effects of food insecurity for postpartum maternal–infant bonding, though we did not find a similar buffering effect for parenting stress. Future research should explore which aspects of social support are most beneficial for pregnant women who have experienced food insecurity; perhaps greater instrumental support is associated with the ability to increase food security.

Limitations of the current study include a small sample size and a predominately low-income and diverse sample, which limit the generalizability of study findings. Despite limitations, these findings provide evidence that the harms of food insecurity can endure over time, such that periconception food insecurity is associated with postpartum parental functioning. Given some evidence of the mediating role of social support, these associations may also reflect persistent food insecurity that parents continue to face postpartum, particularly when they do not have a supportive social network to draw upon.

It is beyond the scope of this study to examine change in food insecurity across the perinatal period, but additional research examining change in food security status is needed to determine whether food insecurity has long term effects (29). It is important to note that both earlier and concurrent food insecurity are associated with suboptimal parenting practices across infancy to age five (30), suggesting that it is critical to prevent any food insecurity to support parent and child well-being. The findings of this study highlight the need for maternal linkages to effective food security programs, such as WIC, during childbearing years due to the critical importance of food security for maternal and infant health and well-being as well as the early mother-infant relationship.

## Data availability statement

The raw data supporting the conclusions of this article will be made available by the authors, without undue reservation.

## Ethics statement

The studies involving humans were approved by Oklahoma State University Institutional Review Board. The studies were conducted in accordance with the local legislation and institutional requirements. Written informed consent for participation in this study was provided by the participants’ legal guardians/next of kin.

## Author contributions

KS: Conceptualization, Data curation, Formal analysis, Methodology, Project administration, Writing – original draft, Writing – review & editing. CD: Writing – original draft, Writing – review & editing. LC: Writing – review & editing. MW: Formal analysis, Writing – review & editing. JC: Visualization, Writing – review & editing.
